# Exposure therapy tailored to inhibitory learning principles in a naturalistic setting: an open pilot trial in obsessive-compulsive outpatient care

**DOI:** 10.3389/fpsyg.2024.1328850

**Published:** 2024-05-13

**Authors:** Franziska Kühne, Lea Kathrin Hobrecker, Peter Eric Heinze, Claudia Meißner, Florian Weck

**Affiliations:** Department of Psychology, Clinical Psychology and Psychotherapy, University of Potsdam, Potsdam, Germany

**Keywords:** anxiety disorders, cognitive behavior therapy, exposure and response prevention, naturalistic study, psychotherapy process

## Abstract

Inhibitory learning (IL) theory offers promising therapeutic strategies. However, more evidence is needed, especially regarding OCD treatment in routine care. The present pilot study investigated the positive and negative effects of IL-focused cognitive-behavioral therapy (CBT) in a university outpatient setting. A total of *N* = 21 patients (57.14% male, mean age 31.14, *SD* = 12.39 years) passed through manualized therapy delivered by licensed psychotherapists. Between the first and 20th IL-focused CBT session, obsessive-compulsive symptoms (Obsessive Compulsive Inventory-Revised, *d* = 3.71), obsessive beliefs (Obsessive-Beliefs Questionnaire, *d* = 1.17), depressive symptoms (Beck Depression Inventory, *d* = 3.49), and overall psychological distress (Global Severity Index, *d* = 3.40) decreased significantly (all *p*s < 0.01). However, individual patients reported some negative effects of therapy. The results underline the value of thorough investigations of novel therapeutic interventions in naturalistic settings.

## 1 Introduction

With a lifetime prevalence of around 1–2% (Kessler et al., 2012; Fawcett et al., 2020), obsessive-compulsive disorder (OCD) is a prevalent mental disorder. It is associated with impairments in a variety of quality-of-life domains, such as work, family, and social life (Stein et al., 2019). Without treatment, a chronic and unremitting course remains likely (Melkonian et al., 2022). As cognitive-behavioral therapy (CBT) is a highly effective form of therapy (e.g., Olatunji et al., 2013; Carpenter et al., 2018; Ferrando and Selai, 2021), it is recommended as a stand-alone intervention, or in combination with psychopharmacotherapy in various treatment guidelines (National Institute for Health and Care Excellence, 2005; American Psychological Association, 2015; German Association for Psychiatry, Psychotherapy and Psychosomatics, 2022). Although exposure with response prevention (ERP), the most prominent component of CBT, leads to a weighted mean dropout rate in OCD of 14.7%, “as a field we would like it to be as low as possible,” which is why Ong et al. (2016) refer to “room for improvement” (p. 15).

In accordance with this statement, researchers advise involving family members in therapy, or augmenting CBT with psychological interventions such as motivational interviewing (Guzick et al., 2018). Both strategies have proven effective, especially for patients with more severe OCD (Guzick et al., 2018). Another promising strategy is to intensify CBT, for instance, through offering at least five (instead of one or two) therapist hours on average per week (Jónsson et al., 2015). Despite the large meta-analytic effects of intensive CBT within short timeframe, mixed results on the maintenance of therapy effects (Jónsson et al., 2015; Kvale et al., 2018) warrant further investigation.

In addition to the above-mentioned strategies for augmenting therapy, ERP informed by inhibitory learning (IL) principles has recently come into the focus of OCD research (Craske et al., 2014; Jacoby and Abramowitz, 2016). The IL theory draws on basic research on fear conditioning and fear extinction, and aims to optimize exposure therapy (for an overview, e.g., Craske et al., 2008, 2014). Its keynotes center around strengthening new learning of non-threatening associations to inhibit older threat associations during ERP, and to improve their retrieval in the long-term after therapy (Craske et al., 2014). Previous empirical investigations on IL focused mainly on specific and social phobias, and found evidence for the effects of specific strategies for fostering IL, namely, expectancy violation and contextual variability (Weisman and Rodebaugh, 2018).

During the last few years, case vignettes for the implementation of IL in ERP were published for the treatment of OCD patients (Craske et al., 2014; Knowles and Olatunji, 2019). Specifically, Jacoby and Abramowitz (2016) narratively reviewed the adaptation of specific strategies to foster IL for OCD treatment. According to the authors, *expectancy violation* (i.e., the maximal violation of expectancies regarding the frequency or intensity of aversive outcomes) may be achieved, for example by asking patients whether their expectations have changed through ERP, or to continue ERP until expectancies have been violated. To further increase expectancy violation, *combining multiple fear cues* could be applied clinically by combining *in vivo*, in *sensu* and/or interoceptive exposure (Jacoby and Abramowitz, 2016). Third, *maximizing contextual* var*iability* not only refers to diversifying the exposure stimuli themselves, but also including the external context (e.g., ERPs alone or in a social situation, at different locations) or including different physiological states during exposures. The last strategy is *expanding the inter-session interval*, such as by gradually tapering off therapy or by offering booster sessions.

However, the current evidence on strategies to promote IL in psychotherapy for OCD patients is still rather narrow, and “there is a need to examine the effects of these rationales on treatment credibility, acceptability, as well as on engagement in exposure and treatment outcome” (Jacoby and Abramowitz, 2016, p. 38). Although IL seems very promising, more empirical research on its benefits and unwanted effects in clinical samples and in routine care is necessary (Jacoby and Abramowitz, 2016; Weisman and Rodebaugh, 2018). Accordingly, we developed IL-focused CBT, and investigated it in the present pilot study. In pilot and feasibility studies, it is necessary to report the risks and potential dangers of a new therapy, for example to inform the design of subsequent trials or to provide information on the future monitoring of potential harm (Eldridge et al., 2016). Thus, we also aimed to investigate the potential negative effects of our therapy. Furthermore, beliefs play a vital role in the development and maintenance of OCD [Obsessive Compulsive Cognitions Working Group (OCCWG), 2005]. Despite not being explicitly tested, they may change during IL focused therapy (Craske et al., 2014), and were therefore also examined.

Against this background, the current open pilot trial aimed to evaluate the impact of the intervention that we had developed, i.e., ERP tailored to IL principles. Specifically, we investigated its effect on the symptomatology of OCD patients treated by licensed psychotherapists in routine outpatient care. In doing so, we investigated whether obsessive-compulsive symptoms decreased over the course of 20 sessions of CBT (primary outcome). Secondly, we investigated whether there was a decrease in obsessive beliefs, in depressive symptoms, and in overall distress during that period (secondary outcomes). Thirdly, we examined the patients’ perceptions, both of their previous psychotherapies (OCD treatment history), as well as the positive and negative effects of the current therapy.

## 2 Methods

### 2.1 Participants

Patients registered for therapy with our outpatient clinic themselves, i.e., they were not referred by another department (see 2.3). Inclusion criteria were a main OCD diagnosis (i.e., ICD-10 F42.0-2) established by means of the Y-BOCS interview (Goodman et al., 1989; Hand and Büttner-Westphal, 1991). The interviews were conducted by a licensed psychotherapist before the start of therapy. Further inclusion criteria were age ≥ 18 years, and sufficient German language skills to follow therapy. Exclusion criteria were a psychotic episode, acute suicidality or substance dependence. Patients were not reimbursed for study participation. Study participation was not associated with any advantages or disadvantages concerning ongoing therapy. As stated in the pre-registered study protocol,^1^ we had planned to consecutively include at least the first 15 patients providing T0 − T20 data.

**Figure 1:**
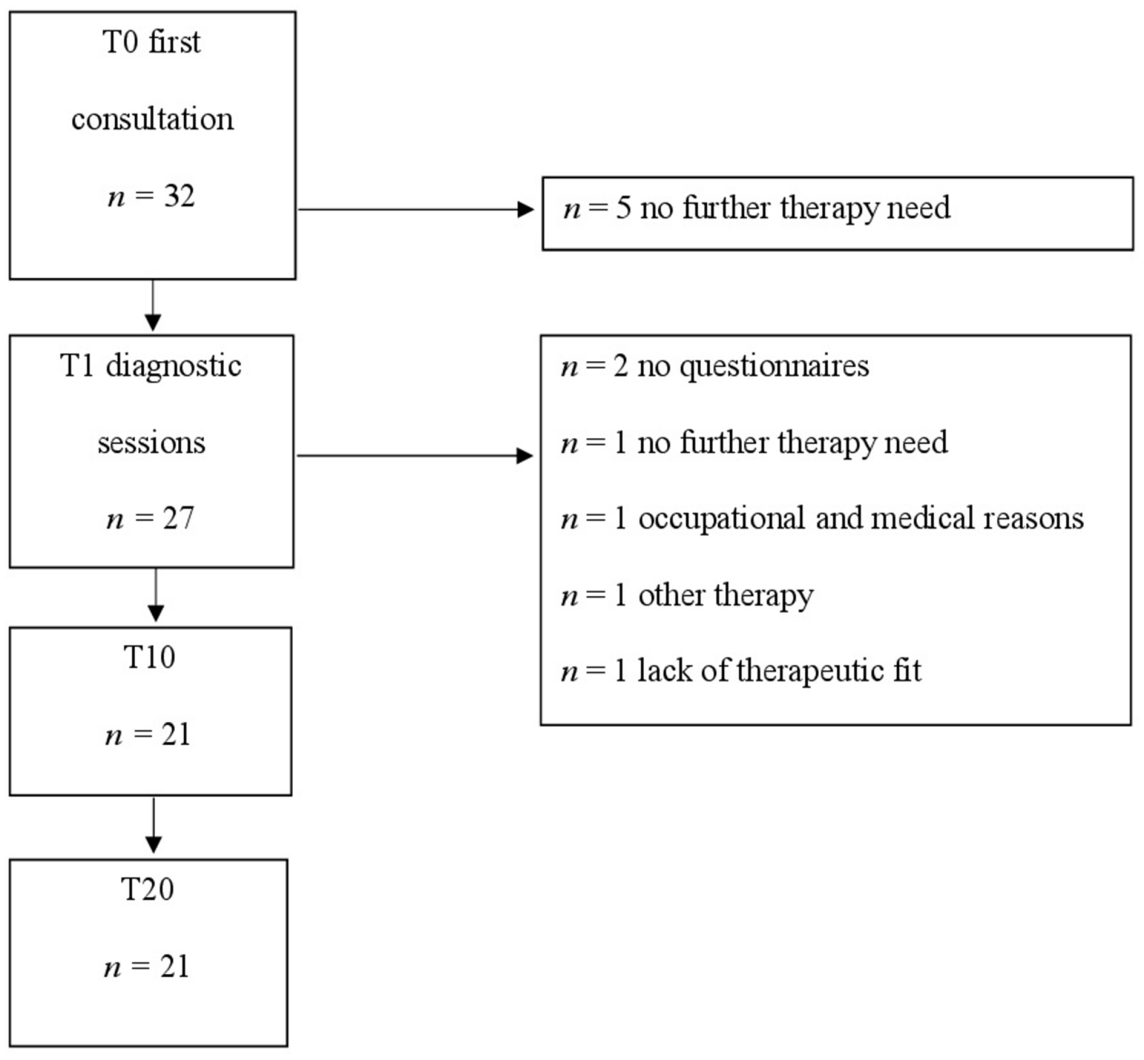
Participant flow.

A total of *N* = 21 patients were enrolled in the present study. Demographic information is displayed in Table 1, for further information regarding study flow, see Figure 1. One Y-BOCS interview was missing. According to Cervin et al. (2022), 9.5% (*n* = 2) of the patients had *subclinical* Y-BOCS values at baseline, the symptom severity of 28.6% (*n* = 6) was *mild*, of 47.6% (*n* = 10) *moderate*, and of 9.5% (*n* = 2) of the patients it was *severe.*

**Table 1:** Participant characteristics (*n* = 21).

Characteristics		*% (n)*
Gender	Male	57.14 (12)
	Female	38.10 (8)
	Not specified	4.76 (1)
Highest education	University degree	33.33 (7)
	High school diploma	33.33 (7)
	Lower secondary education	14.29 (3)
	No degree	9.52 (2)
	Secondary school	4.76 (1)
	Other	4.76 (1)
Nationality	German	95.24 (20)
	French	4.76 (1)
Psychotropic medication^1^	SSRI	23.81 (5)
	Stimulants	14.29 (3)
	Neuroleptic drugs	9.52 (2)
Comorbid mental disorder^1^	Other anxiety disorder	33.33 (7)
	Depressive disorder	14.29 (3)
Age (in years)^2^	31.14 ± 12.39, 18–70	
Number of therapy sessions^2^	34.86 **±** 14.44, 18–60	

^1^*n* = 10 indications, ^2^M ± SD, Range.

According to the clinical interviews (see Procedure), *n* = 6 patients received comorbid diagnoses of an anxiety disorder (agoraphobia, social phobia, hypochondriasis, specific phobia, and other), and *n* = 3 patients were diagnosed with a comorbid depressive disorder.

### 2.2 Measures

#### 2.2.1 Primary outcome

##### 2.2.1.1 Obsessive compulsive inventory-revised

The 18-item Obsessive Compulsive Inventory-Revised (OCI-R) measures six common OCD symptom domains (Foa et al., 2002; Gönner et al., 2008). Its items are rated on a five-point scale (from 0 = *not at all* to 4 = *very strong*), the sum scores range between 0 and 72. Using the total score, OCD symptoms can be classified as *mild* (0–15), *moderate* (16–27), or *severe* (28–72; Abramovitch et al., 2020). In the present study, the OCI-R showed good internal consistency (Cronach’s *α* = 0.82), which is in line with Abramovitch et al. (2020) (*α* = 0.83).

#### 2.2.2 Secondary outcomes

##### 2.2.2.1 Obsessive-beliefs questionnaire

The Obsessive-Beliefs Questionnaire (OBQ) measures dysfunctional beliefs on the following subscales: (1) the importance of thoughts and the need to control thoughts, (2) overestimations of threat and inflated personal responsibility, and (3) perfectionism and intolerance of uncertainty [Obsessive Compulsive Cognitions Working Group (OCCWG), 2005; Ertle et al., 2008]. The OBQ consists of 24 items answered on a seven-point scale (from 1 = *disagree very much* to 7 = *agree very much*). We used the total score, with higher values indicating greater agreement with OCD-related dysfunctional beliefs. In the current study, the instrument’s internal consistency was good (*α* = 0.96). For the measurement times, see Table 2.

**Table 2:** Baseline symptoms (*n* = 21), measurement times, and symptoms in the course of treatment.

	*M_T1_*	*SD_T1_*	*Range_T1_*	*Interpretation_T1_*	*M_T10_*	*SD_T10_*	*M_T20_*	*SD_T20_*
OCI-R	22.71	11.35	8–48	Moderate	18.14	10.33	14.25	11.07
Y-BOCS-Int^1^	21.70	5.76	11–31	Mild to moderate	-	-	-	-
BDI-II	15.90	9.90	1–34	Mild	11.95	7.04	9.95	7.89
OBQ^1^	4.13	1.47	1.29–6.58	Comparable to OCD patients^2^	-	-	3.34	1.54
GSI	1.04	0.67	0.09–2.55	Below cutoff	45.50	27.09	39.17	26.68

OCI-R, Obsessive-Compulsive Inventory-Revised; Y-BOCS-Int, Y-BOCS interview; BDI-II, Beck Depression Inventory-II; OBQ, Obsessive-Beliefs Questionnaire; GSI, Global Severity Index; ^1^*n* = 20; ^2^n.s. difference to OCD sample (*p* = 0.496; Ertle et al., 2008).

##### 2.2.2.2 Beck depression inventory

The Beck Depression Inventory (BDI-II) measures the severity of depressive symptoms by means of 21 items (Beck et al., 1996; Hautzinger et al., 2009). Each item represents a symptom of the affective, cognitive, motivational, vegetative, and psychomotor components of depression. Using the total score (ranging from 0 to 63), depressive symptoms can be classified as *minimal* (9–13), *mild* (14–19), *moderate* (20–28), or *severe* (> 29; Hautzinger et al., 2009). The BDI-II showed good internal consistency in our study (*α* = 0.91).

##### 2.2.2.3 Global severity index

The Global Severity Index (GSI) is a composite score of the Brief Symptom Inventory (BSI; Derogatis, 1982; Franke, 2017) and a global index of overall psychological distress (e.g., anxiety, somatization). The BSI consists of 53 items, answered on a five-point scale (from 0 = *not at all* to 4 = *very strong*). Its internal consistency was good in the present study (*α* = 0.96). The GSI is obtained by dividing the total BSI score by the number of answered items. The level of psychological distress can be classified as clinically relevant from the cutoff point of a *t*-value over 60 for a given GSI score (Franke, 2017).

#### 2.2.3 Perception of previous psychotherapies

##### 2.2.3.1 OCD-treatment history questionnaire

We had previously translated the OCD-Treatment History Questionnaire (OCD-TH) into German (Marschner et al., 2019). Its 19 items relate to experiences with previous treatments (e.g., “Spent most of the sessions talking about my childhood/past experiences”), and are answered with “yes” or “no.” In the event of a positive answer, participants state on a six-point Likert-scale from --- to +++ how helpful they found a certain treatment to be (Stobie et al., 2007). Participants filled out the OCD-TH at the beginning of treatment (T1).

#### 2.2.4 Positive and negative effects of current therapy

##### 2.2.4.1 Positive and negative effects of therapy scale

The Positive and Negative Effects of Therapy Scale (PANEPS) consists of 29 items initially rated on a four-point scale (from 0 = *applicable*, 1 = *rather applicable*, 2 = *rather not applicable*, 3 = *not applicable*, Peth et al., 2018). We then dichotomized the items, i.e., if participants answered with *applicable/rather applicable*, we coded their answer using 1, otherwise using 0. Participants filled out the PANEPS at the end of treatment (T20). In the present study, the internal consistency was acceptable to excellent for all subscales (Side Effects *α* = 0.67, Malpractice *α* = 0.91, Unethical Conduct *α* = 1.00), except for Positive Effects (α = 0.09). Thus, we did not use the latter subscale for further analysis.

### 2.3 Procedure

The study is an open pilot trial in a naturalistic setting including three measurements (T1, T10, and T20). Participants were recruited at the University of Potsdam’s psychotherapy outpatient clinic, between 12/2017 and 08/2021. Before inclusion in the study, they provided informed consent (i.e., permission to use their pseudonymized clinical and sociodemographic data for research). Ethical approval for conducting research in our outpatient clinic was granted by the University of Potsdam’s ethics committee (no. 73/2016). There was no public advertisement for the study to recruit patients. Patients who started psychotherapy were informed about the study by the treating therapist.

After patient registration, one of two licensed psychotherapists not involved in data analysis conducted the 1-h entry interview face-to-face with the patient. In the following diagnostic sessions, the same therapist conducted the Y-BOCS interview (Hand and Büttner-Westphal, 1991), and the International Diagnosis Checklists for ICD-10 (IDCL) clinical interview (Hiller et al., 1995). Patients then waited for *M* = 13.1, *SD* = 8.89 weeks for their diagnostic sessions with their treating therapists. These sessions precede psychotherapy according to the German care system. This was followed by CBT with the treating therapist.

### 2.4 Treatment

At our outpatient clinic, we provide evidence-based treatments mainly for patients diagnosed with anxiety and obsessive-compulsive disorders, using CBT as recommended in national treatment guidelines (e.g., German Association for Psychiatry, Psychotherapy and Psychosomatics, 2021, 2022). Assuming sufficient capacity, we also treat with CBT, patients with all other mental disorders.

In order to tailor individual therapy to inhibitory learning principles, we developed a treatment manual conceptualized as short-term therapy according to German law (i.e., 24 sessions à 50 min, financed by a German health insurance if a mental disorder was formally diagnosed, and if no other outpatient psychotherapy was made use of during the last 2 years). The treatment manual was based on previous literature (Hand and Büttner-Westphal, 1991; Emmelkamp and van Oppen, 2000; Research Consortium on Intrusive Fear, 2007; Wahl et al., 2007; Ertle, 2012; Pittig et al., 2015; Jacoby and Abramowitz, 2016; Lakatos and Reinecker, 2016; Weck, 2017; Abramowitz, 2018; Salkovskis et al., 2018), and comprised three modules:

- Psychoeducation (Information on OCD, Development and maintenance, and Therapy goals).
- Exposure with response prevention (Behavioral and thought experiments, ERP, and Self-management).
- Relapse prevention (Relapse signs, Helpful contacts, Aims for the future, and Booster sessions)

Therapists were given examples of phrases they could use with their patients (e.g., “*Paradoxically, the way to less obsession is through learning to tolerate them more openly, and through accepting uncertainty – a skill that develops through practice like playing an instrument or riding a bike*”, based on Abramowitz, 2018, p. 69). Difficulties (e.g., neutralizing behavior after completed ERP) were addressed, and examples of how to deal with them were given proactively. Treatment was based on behavioral principles; thus cognitive interventions were used for psychoeducation or motivation only. Acceptance-based metaphors were integrated in order to increase distress tolerance or to illustrate the function of thought repression only.

The manual addressed IL principles such as *expectancy violation, maximizing contextual* var*iability, combining multiple fear cues* and *expanding the inter-session interval* as described above and in Supplementary material 1. Importantly, therapists were instructed that habituation was not the only, and also not a necessary exposure goal. In that respect, the end of ERP was defined by various mechanisms, e.g., by coping successfully with the situation, by learning something new or by perceiving habituation. The first ERPs were therapist-led, homework was given from the first therapy session onwards. In addition, each patient received a workbook with worksheets to complete individually at home or during therapy. Patients were informed as to the active nature of therapy, about possible positive and negative effects, and about the number of sessions planned. To prevent allegiance effects, they did not receive information on the novelty of our IL-focused intervention.

Although therapy was performed once weekly (50 min. each), longer or more frequent sessions were used for therapist-led exposures in the patients’ everyday environment. The last four sessions were planned as booster sessions, and due to intervals differing between patients, were not part of the current data analysis. Therapy was implemented by four psychotherapists with a CBT licensure according to German law. All had university degrees (Master or the German “Diplom”) as clinical psychologists, and subsequently received three to 5 years of formal training in CBT. They thus had 5, 6, 8, and 11 years of professional experience, and had, respectively, been licensed for 1, 2, 3, and 6 years at the beginning of recruitment. All therapists received a 1-day training session on how to implement the treatment manual.

Therapy was modularized, and, based on the individualized treatment plan, therapists decided on the length of interventions, on the omission of specific components if not deemed necessary (e.g., thought experiments), and to extend CBT beyond short-term therapy if necessary and justified. Therapists received supervision on using the manual every 4–6 weeks by the first author. Treatment adherence was reported by the therapists on a separate form, and analyzed by the second author, who was not part of the therapeutic team. Only minor deviations occurred (e.g., that relapse prevention was not an issue, due to ongoing therapy, or that an intervention from Acceptance and Commitment Therapy was used).

### 2.5 Data analysis

Statistical analyses were performed using IBM SPSS Statistics 29.0 and Microsoft Excel. Missing data concerned entire questionnaires (i.e., two persons did not fill out the T10 questionnaires, and one did not fill out the T1 or T20 questionnaires, respectively). Therefore, missing data were not imputed. The alpha level was set at 0.05, but corrected for multiple testing, using Bonferroni correction for analysis of the secondary outcomes. We used general linear models (GLMs) and paired *t*-tests for dependent samples to investigate symptom change over time, and provide effect sizes (i.e., *d* ≥ 0.80 as *large*; Cohen, 1988).

Regarding the primary OCD outcome (i.e., the OCI-R), we calculated the Reliable Change Index (RCI; Jacobson and Truax, 1991; Supplementary material 1). Based on previous research, we defined treatment response as a ≥ 40% reduction in total OCI-R score from pre-treatment, and remission as an OCI-R post-treatment score of ≤8 (Flygare et al., 2023). Deterioration was defined as a significant symptom increase from T1 to T20 (i.e., +1.96; Jacobson and Truax, 1991). All other patients were then classified as unchanged.

Descriptive statistics were used to examine demographic information, the OCD treatment history, and the perceived positive and negative effects of therapy. With a sample size of *n* = 19 OCI-R measurements, we had sufficient power (0.85) to detect medium effects (*f* = 0.25) between T1 and T20 for one group, three measurements, and the present large correlation between repeated measurements (*r*_T1–T20_ = 0.71; G*Power 3.1.9.4; Faul et al., 2007).

## 3 Results

### 3.1 Change of OCD symptoms over treatment

Descriptive statistics for the primary and secondary measures (T1) are presented in Table 2. Obsessive-compulsive symptoms (OCI-R) decreased significantly over time [*F*(1,17) = 58.578, *p* < 0.001, *d* = 3.71]. Symptoms were less severe, but still *moderate* at T10, and could be classified as *mild* at T20 (Table 2).

Referring to the RCI, 40% (*n* = 8) of the patients achieved reliable change. Thus, 60% (*n* = 12) remained unchanged until T20, while no patient deteriorated reliably. Defining treatment response as a ≥ 40% OCI-R reduction, and remission as an OCI-R post-treatment score of ≤8 (Flygare et al., 2023), 30% (*n* = 6) of the patients responded, and 25% (*n* = 5) were remitted. According to clinical assessment, including patient preferences, and following education about the positive and negative effects of (dis)continuation of therapy, CBT was often continued. Half of the patients received short-term therapy (i.e., 18–26 sessions), and the other half completed long-term therapy (i.e., up to 60 sessions according to German regulations).

### 3.2 Change of secondary outcomes over treatment

Between T10 and T20, obsessive beliefs (OBQ) declined significantly (*T* = 3.115, *p* = 0.009, *d* = 1.17). Depressive symptoms (BDI-II) also decreased significantly [*F*(1,18) = 54.910, *p* < 0.003, *d* = 3.49], and were *minimal* at T10 and at T20. Likewise, there was a significant decline in psychological distress [GSI; *F*(1,17) = 49.205, *p* < 0.003, *d* = 3.40], whereas both the T10 and T20 means were below the clinical cutoff (Table 3).

**Table 3:** Perception of previous psychotherapies and negative effects of current therapy.

Shortened OCD-TH items (item number)	*n*	Mean helpfulness^1^ (*SD*)
Explored childhood to better understand the present (6)	14	4.50 (1.02)
Looked at links between beliefs, thoughts, and feelings (7)	13	4.46 (0.88)
Got homework between sessions (19)	12	4.58 (1.08)
Changed the meaning attached to thoughts (18)	12	4.50 (1.00)
Silent therapist for most of the sessions allowed to talk freely (11)	12	4.67 (1.07)
Exposure in the therapist’s office (4)	12	4.83 (0.84)
Concentrated on beliefs about obsessions in most sessions (10)	11	4.09 (0.83)
Most of the sessions on childhood/past experiences (3)	11	4.55 (0.82)
Explored recurrent patterns of relating (8)	10	3.30 (1.16)
Received or worked on reading material on the problem (12)	10	4.40 (1.43)
Changed behavior rather than working directly on thoughts (14)	9	4.00 (1.12)
Faced situations outside of the therapy room without therapist (5)	8	4.13 (0.99)
Drew a diagram explaining the problem (1)	6	4.50 (1.05)
Kept thought records (13)	6	3.83 (1.47)
Did relaxation exercises (17)	6	4.17 (1.33)
Mostly looking at problems other than the obsessional problems (16)	6	3.67 (1.03)
Exposure outside the therapy room together with the therapist (9)	5	3.40 (2.30)
Drew a diagram showing patterns of relationships (2)	4	4.00 (0.82)
Tried techniques to stop thoughts (15)	3	4.00 (1.00)
Negative effects of current therapy (PANEPS subscales)		Mean affirmation^2^ (*SD*)
Side effects		0.12 (0.17)
Malpractice		0.22 (0.10)
Unethical conduct		0 (0)

^1^Multiple indications allowed; From --- (1) to +++ (6); ^2^Rather/applicable.

### 3.3 Individual perception of previous and current therapy

Concerning their previous psychotherapy, patients mostly did not remember specific interventions (Table 3). If they reported previous interventions, they were very diverse, but were perceived overall as helpful. In line with this, 33.33% (*n* = 7) patients reported previous CBT, 28.57% (*n* = 6) psychodynamic therapy, and 4.76% (*n* = 1) psychoanalytic therapy.

Regarding current psychotherapy, some patients perceived side effects (Table 3). Single items were affirmed by max. *n* = 5 patients (such as “*My application for private insurance was denied because I was doing therapy*” or “*I am afraid that my social environment will find out that I am in therapy*”). The five individual indications of malpractice were all given by the same patient (e.g., “*I had the feeling that my therapist did not like me*”). No patient described having experienced unethical conduct.

## 4 Discussion

Within the current pilot study, we investigated the effect of exposure therapy tailored to IL principles in outpatient psychotherapy. First of all, obsessive-compulsive symptoms, obsessive beliefs, depressive symptoms, and overall psychological distress decreased significantly between the first and the 20th CBT session, each with large effects. Our findings are in line with the results of a large naturalistic effectiveness study of CBT in OCD patients in a similar care setting, which also found large treatment effects (Kathmann et al., 2022). However, at T20, patients on average still perceived mild obsessive symptoms, 60% did not achieve any reliable symptom change, only 25% were remitted, and 50% continued with long-term psychotherapy. Comparably, the OCD patients from the large German naturalistic study received 50 CBT sessions on average, and the symptoms of around 40% of the patients did not change reliably until their end of therapy (Kathmann et al., 2022).

Altogether, CBT is a highly effective psychotherapy for OCD (e.g., Olatunji et al., 2013; Carpenter et al., 2018) also in routine care (Öst et al., 2022). However, extending therapy does not necessarily increase its output, as the number of therapy sessions was not a significant moderator of therapy effects in previous meta-analyses (Olatunji et al., 2013; Ferrando and Selai, 2021; Öst et al., 2022). Therefore decisions, for example on the continuation of therapy, should not be based on clinical reasoning alone, but complemented with routine outcome monitoring that is immediately fed back to therapists, especially for patients with a high probability of poor therapy outcome (Lambert et al., 2018).

Our major objective was to contribute to the evidence base regarding IL-focused CBT in routine care. As the description of our therapy concept shows, CBT and IL were interrelated, which hampers dismantling effects. However, from a theoretical point of view, IL and habituation are not mutually exclusive, but additional processes underlying fear extinction (Craske et al., 2014). Habituation can be assumed an indicator of change (Jacoby and Abramowitz, 2016) that, according to IL theory, may or may not occur. Accordingly, from a clinical point of view, there are difficulties distinguishing habituation-based from IL-based procedures (Sauer and Witthöft, 2022). As Weisman and Rodebaugh (2018, p. 36) put it, IL should thus be regarded “an overarching way to conduct exposure,” “a unified exposure paradigm, with dual foci on violating expectancies and incorporating variability,” and thereby an “ideal path to improving the efficacy of exposure.” In addition, there are further important mechanisms of change, such as increased distress tolerance, or improved self-efficacy, which also contribute to the effectiveness of exposure (e.g., Knowles and Olatunji, 2019; Sauer and Witthöft, 2022).

As the patients’ answers to the OCD-TH might suggest, most patients did not remember their previous therapy in detail (i.e., between three and 14 patients responded to the items), but those who did so were often relatively content with the interventions (i.e., all means were in the positive range). However, previous therapies were not remembered as delivered, according to the current treatment guidelines. In our study, no patient deteriorated reliably, but on a descriptive level, two had higher OCI-R values at session 20. Furthermore, around five patients perceived single side effects of therapy, and one patient was particularly discontent, reporting individual forms of malpractice. It is a strength of our study that we addressed negative effects of therapy explicitly, whereby it became clear that the problems of one particular patient were not considered sufficiently within therapy. Again, immediate feedback after each session (Lambert et al., 2018) could have contributed to reducing negative effects.

### 4.1 Limitations

While the naturalistic study design is a strength of our study, the absence of a control group, lack of follow-up and the small sample size of the open trial are major limitations. In addition, future studies on IL-focused CBT should compare intention-to-treat with completer samples, include Y-BOCS interviews conducted by independent clinician’s pre and post therapy, use the Y-BOCS self-report checklist to depict the severity of OCD symptoms, and conduct adherence checks through the investigation of therapy videos by independent research staff. Despite its limitations, the current pilot study offers useful empirical results on the role of IL-focused CBT in routine care. It also provides indications of how difficult it may be to depict potentially small differences in a family of interventions (i.e., CBT) in routine care. Therefore, future studies should use measures of patient acceptance and subjective appraisal of the IL components of therapy.

### 4.2 Conclusion

According to our study, CBT was effective, but it remains an open empirical question how to address those patients who do not benefit sufficiently. IL-focused therapy was hardly distinguishable from CBT as implemented in practice. Thus, future studies should compare more clearly-defined intervention groups, and investigate the acceptance and effectiveness of the IL components of therapy more directly. Furthermore, negative effects of therapy should be reported as standard, in order to avoid memory and other bias.

## Data availability statement

The datasets presented in this article are not readily available because of the present ethics vote. Requests to access the datasets should be directed to dr.franziska.kuehne@uni-potsdam.de.

## Ethics statement

The studies involving humans were approved by the University of Potsdam’s ethics committee (no. 73/2016). The studies were conducted in accordance with the local legislation and institutional requirements. Written informed consent for participation in this study was provided by the participants’, or their legal guardians/next of kin.

## Author contributions

FK: Conceptualization, Formal analysis, Investigation, Methodology, Project administration, Validation, Visualization, Writing – original draft. LH: Data curation, Validation, Writing – review & editing. PH: Data curation, Methodology, Validation, Writing – review & editing. CM: Data curation, Writing – review & editing. FW: Resources, Software, Supervision, Writing – review & editing.

## Footnotes

^1^ osf.io/gtx3u
