# Supplementary material for: Exposure therapy tailored to inhibitory learning principles in a naturalistic setting: an open pilot trial in obsessive-compulsive outpatient care
Source: Front Psychol. 2024 May 13;15:1328850. doi: 10.3389/fpsyg.2024.1328850 (PMC11129681; doi:10.3389/fpsyg.2024.1328850)
Supplement: Supplementary file 1 [file Data_Sheet_1.PDF]

Supplement 1

Implementation of IL strategies in the current treatment

**IL strategy**	**Examples from the manual**
Expectancy violation	– What did patients discover and learn during the exercise?<br>– What surprised them?<br>– Were there any discrepancies between their expectations and what has occurred?
Maximizing contextual variability	– Guide the patient to practice in different settings and situations and in different ways<br>– e.g. at home, at work, in public, alone, in the presence of others, with/without conversations, with/without music, etc.
Combining multiple fear cues	– Guide the patient to use a random selection of situations for exposures with different levels of difficulty<br>– Combine in vivo with in sensu and interoceptive exposures (e.g., run the stairs before)<br>– Perform exposures at different levels of excitement (relaxed or stressed)
Expanding the inter-session interval	– Use sessions 21-24 as booster sessions (after 1, 2, 3 and 6 months)<br>– Have OCD symptoms appeared? Which situations was s/he able to cope with (less) well? What can the patient learn from this?

Calculation of the Reliable Change Index (Jacobson & Truax, 1991)

$$SE = 11.35\sqrt{(1 - .82)} = 4.82$$

$$Sdiff = \sqrt{2\,(4.82)^2} = 6.82$$

$$RC = \frac{x\,(T20) - x\,(T1)}{6.82}$$

Deviations from the study protocol (osf.io/gtx3u)

In the protocol, we had planned to impute missing data by their individual mean. However, in the study missing data concerned entire questionnaires, and not single values, which is why we did not impute them.

In the protocol, we had planned to use the Dimensional Obsessive-Compulsive Scale-short forms (DOCS-SF, Kühne et al., 2021) as a primary outcome measure. However, we were able to include the questionnaire into our test battery for the last few patients only, which is why we do not report the results here.

Furthermore, we had planned to use the Y-BOCS symptom checklist as a self-rating instrument. The measurement time points of the present study were T1, T10, and T20. As the symptom checklist was included at T0 (first consultation) only, but not at T1 one to seven months later, we did not use it for the current analysis.
